# Study of Antiphospholipid Antibodies in Patients with Arterial Hypertension

**DOI:** 10.3390/medsci6040102

**Published:** 2018-11-13

**Authors:** Gioulia Romanidou, Theocharis G. Konstantinidis, Odysseas Koutsogiannis, Anastasia Grapsa, Konstantina Kantartzi, Stylianos Panagoutsos, Maria Panopoulou, Christina Tsigalou

**Affiliations:** 1 Department of Nephrology, Democritus University of Thrace, University General Hospital of Alexandroupolis Dragana Campus, 68100 Alexandroupolis, Greece; dr_giouliarom@yahoo.gr (G.R.); kokan0910@gmail.com (K.K.); spanagou@med.duth.gr (S.P.); 2 General Hospital “Sismanoglio”, Sismanoglou 45, 69133 Komotini, Greece; theoxari_ko@yahoo.gr; 3 Laboratory of Microbiology, Democritus University of Thrace, University General Hospital of Alexandroupolis, Dragana Campus, 68100 Alexandroupolis, Greece; anastasiagrapsa@yahoo.gr (A.G.); mpanopou@med.duth.gr (M.P.); xtsigalou@yahoo.gr (C.T.); 4 Health Centre of Iasmos, 69200 Iasmos, Greece

**Keywords:** antiphospholipid antibodies, hypertension, neutrophil gelatinase associated lipocalin (NGAL)

## Abstract

Antiphospholipid syndrome (APS) is a multifactorial, autoantibody-mediated disease. Antiphospholipid antibodies (aPL) directed against negatively charged phospholipids or various combinations of phospholipid-binding proteins seem to be an independent pathogenic factor that plays a critical role in APS. Unfortunately, their role in hypertension is not fully elucidated. The aim of our study was to determine aPL titers in hypertension patients and investigate the association of aPL with renal impairment parameters. Forty-seven patients with arterial hypertension (22 males, 46.8% and 25 females, 53.2%), aged 41–85 years old (mean 65.9 ± 10.1 years), and 21 age-sex-matched subjects without severe hypertension as control group (8 males, 13 females, 38.1% vs. 61.9%), mean age 61 ± 11.3 years, were enrolled in this study. Patients with other risk factors like Rheumatoid Arthritis and Systematic Lupus Erythematosus (SLE), both viral and bacterial acute infections, and cancer were excluded from the study. The aPL (anticardiolipin (ACA) and anti-b2GPI antibodies, IgG and IgM) were measured by ELISA (Aesculisa, Aesku Diagnostics, Wendelsheim, Germany) with a cutoff of 15 GPL/MPL for ACA and 15 U/mL for b2GPI. Serum Neutrophil gelatinase-associated lipocalin (sNGAL) was measured by ELISA kits (BioVendor, Brno, Czech Republic). Biochemical analysis such as serum creatinine (Cr), were measured by automated analyzer and finally estimated glomerular filtration rate (e-GFR) was calculated by the Chronic Kidney Disease Epidemiology Collaboration (CKD-EPI). Fifteen patients were positive for ACA IgG (31.9%), two for anti-b2GPI IgM (4.2%), and three for anti-b2GPI IgG (6.3%). Furthermore, three persons from control group were positive in anti-b2GPI IgG (14.27%). The serum level of anti-b2GPI IgG was significantly higher in patients compared to healthy controls (*p* = 0.013). The level of sNGAL (59.63 ± 41.5 ng/mL vs. 45.5 ± 21.5 ng/mL, *p* = 0.14) was not higher in hypertensive patients than in the age-sex-matched control group. Additionally, the sNGAL level was found to be directly and positively correlated in patients with positive ACA IgG (r^2^ = 0,945, *p* < 0.0001). These results demonstrate that autoimmunity may be one of the pathogenetic factors of hypertension and aPL antibodies might be a potential marker of renal involvement.

## 1. Introduction

The term antiphospholipid syndrome (APS) was coined by Hughes in the early 1980s to describe a unique form of autoantibody induced thrombophilia, hallmarks of which are recurrent thrombosis and pregnancy complications [1]. Nowadays, it is well known that APS is a multisystem autoimmune disorder characterized by an increased risk of vascular thrombosis, pregnancy complications, and a prevalence of autoantibodies called antiphospholipid antibodies (aPL). These aPL are a heterogeneous group of autoantibodies directed against negatively charged phospholipids (such as cardiolipin), protein-phospholipid complexes, or plasma proteins such as β 2-glycoprotein I (b2GPI) [2]. Recently, it was shown that aPL are also present in non-thrombotic diseases such as diabetic nephropathy [3].

Antiphospholipid syndrome may be presented with many other clinical symptoms that are not included in the classification criteria (Table 1). Less common manifestations of APS are heart valve disease, coronary artery disease, hemolytic anemia and HELP syndrome (Hemolysis, Elevated Liver enzymes and Low Platelet count syndrome) [4,5]. The kidney is one of the target organs in APS. The renal involvement is associated with both primary and secondary APS [6]. The well-recognized renal features of APS are renal artery thrombosis/stenosis, renal infarction, renal vein thrombosis, end-stage renal disease (ESRD), and a small-vessel vaso-occlusive nephropathy recently defined as APS nephropathy [7]. Except for thrombotic manifestations, renal involvement is characterized by massive proteinuria and hypertension [8]. Hypertension may be the first clinical symptom of Primary APS (p.APS) and in secondary APS [9]. A relationship between aPL and hypertension is recognized and has recently been discussed by Nasonov et al. and Nzreue et al. [10,11]. According to other previous studies, hypertension may be one of the clinical characteristics in APS patients with renal disease, not all APS patients. Moreover, blood pressure is difficult to control suggesting a renovascular mechanism [12].

Renal dysfunction is definitely a very complicated situation, and this is the main reason for continuing investigation in this field. Renal function is mainly assessed by creatinine-based estimated glomerular filtration rate (e-GFR) equations. According to The Kidney Disease: Improving Global Outcomes (KDIGO) 2012 [13], Chronic Kidney Disease (CKD) is defined by markers of kidney damage (i.e., albuminuria, histological abnormalities) or GFR < 60 mL/min/1.73 m^2^. The different stages of CKD form a continuum. The stages of CKD are classified as follows:

- Stage 1: Kidney damage with normal or increased GFR (>90 mL/min/1.73 m^2^)
- Stage 2: Mild reduction in GFR (60–89 mL/min/1.73 m^2^)
- Stage 3a: Moderate reduction in GFR (45–59 mL/min/1.73 m^2^)
- Stage 3b: Moderate reduction in GFR (30–44 mL/min/1.73 m^2^)
- Stage 4: Severe reduction in GFR (15–29 mL/min/1.73 m^2^)
- Stage 5: Kidney failure (GFR < 15 mL/min/1.73 m^2^ or dialysis)

Patients in stages 1–3 are usually asymptomatic. Thus, there is a great need for novel biomarkers aimed at early and accurate diagnosis of diminished kidney function to prevent rapid deterioration and progressive renal injury.

One of the most studied and promising biomarkers is NGAL, a small molecule released in blood or urine. Potential situations leading to elevated GFR are Acute Kidney Injury (AKI), tubular cell injury, or inflammation. Researchers have confirmed that a low stable secretion maintains serum levels of around 20 ng/mL. Different diseases such as neoplasia, sepsis, CKD, pancreatitis etc. may be the trigger for epithelial impairment and subsequent augmented production [14]. Serum creatinine (sCr) remains the gold standard with regards to kidney failure. On the other hand, NGAL is considered a very promising marker for the early detection of structural kidney dysfunction.

The aim of our study was to evaluate aPL in a group of hypertensive patients with severe essential hypertension and estimate the renal function compared to a control group. Renal dysfunction, as measured by reduced GFR and/or increased urinary albumin excretion, revealed possible association with hypertension. The novel serum marker NGAL was also used to highlight renal dysfunction.

## 2. Materials and Methods

This case-control study included 47 patients with hypertension from outpatient clinics, not selected by sex or ethnicity. Hypertension was diagnosed following the recommendation of the World Health Organization/International Society of Hypertension (World Health Organization, International Society of Hypertension Writing Group, 2003). It is generally accepted that the presence of aPL is associated with acute bacterial and viral infection, neoplasms, ESRD, and acute cardiovascular diseases such as myocardial infarction. This may lead to transient and asymptomatic increase of aPL antibodies or may be an important step during antiphospholipid syndrome. The exclusion criteria were as follows: (1) recent acute myocardial infarction; (2) acute ischemic stroke; (3) neoplasms (current or past); (4) acute viral or bacterial infection; (5) autoimmune disorders such as SLE; (6) ESRD. Moreover, no patient had a history of pregnancy-morbidity such as unexplained fetus death. Twenty-one age and sex-matched non-hypertensive subjects were studied as controls.

Clinical and demographic data were obtained from an interview with patients, considering the following information: (1) age, sex, race/ethnicity; (2) history of high blood pressure; (3) current cigarette smoking; (4) Diabetes Mellitus (DM), according to clinical history and current treatment with oral antidiabetic drugs and/or insulin; (5) history of hypercholesterolemia. All data are shown in Table 2.

Systolic and diastolic blood pressures (SBP and DBP, respectively) were measured while the patients were at rest in a sitting position by an aneroid sphygmomanometer. The hypertensive condition was confirmed when SBP rose to 140 mmHg or more, or DBP elevated to 90 mmHg or more.

Serum samples were centrifuged and frozen within 2 h of collection and subsequently stored at −70 °C until testing by different assays. The aPL, both IgG and IgM types (ACA and anti-b2GPI), were measured by ELISA (Aesculisa, Aesku Diagnostics, Wendelsheim, Germany) with dilution of 1:100 and concentration above 15 GPL/MPL for all isotypes constituting a positive result. Positivity of tests was considered based on proposed values from the manufacturer, due to its agreement with the 99th percentile analysis of 30 blood donors.

To estimate the renal function sCr was measured using an automated biochemical analyzer, and the estimated glomerular filtration rate (e-GFR) was calculated using CKD EPI formula. Finally, sNGAL levels were also measured by ELISA commercial kits (BioVendor, Brno Czech Republic).

The study was approved by the Bioethics Committee of the University General Hospital of Alexandroupolis, Greece (Ethics Committee identification code: 941).

### Statistical Analysis

Data are expressed as mean ± standard deviation and percentages were analyzed. Nonparametric tests (Mann-Whitney and Kruskall-Wallis) and the chi-square (*χ*^2^) test were performed. A value of *p* < 0.05 was considered as statistically significant. The correlation between two quantitative variables was determined with the Pearson’s correlation tests.

## 3. Results

### 3.1. Patients

Group A included 47 patients (22 males, 46.8% and 25 females, 53.2%), aged 41–85 years old (mean 65.9 ± 10.1 years). Of these patients 14 (29.8%) were diabetic, 9 (19.1%) had a history of Acute Coronary Syndrome (ACS), and 70.2% had dyslipidemia. Body mass index (BMI) was 29.7 ± 6.4 kg/m^2^ (Table 2). Group B included 21 control subjects without severe essential hypertension (8 males, 13 females, 38.1% vs. 61.9%), mean age was 61 ± 11.3 years. Among these, 4.7% were diabetic, 33.3% had dyslipidemia, and no patients had a history of ACS. Body mass index was 29.5 + 4.1 kg/m^2^ (Table 2).

### 3.2. Serum Level of aPL

Levels of ACA >15 GPL/MPL and b2GPI >15 U/mL were considered positive (for IgG, or IgM), according to the manufacturer’s guidelines. Among these, 15 were positive for ACA IgG (31.9%), two for anti-b2GPI IgM (4.2%) and three for anti-b2GPI IgG (6.3%). Furthermore, three persons from control group were positive for anti-b2GPI IgG (14.27%), but the mean serum level of anti-b2GPI IgG was not different between patients and controls (16.5 vs. 15.07, *p* = 0.68). The mean serum level of anti-b2GPI IgG was significantly higher in patients in comparison to healthy controls (5.3 ± 5.9 vs. 4.42 ± 1.1, *p* = 0.013). The mean levels of ACA (IgG and IgM) and anti b2GPI IgM were not different between controls and patients (Table 3).

### 3.3. Measurement of Serum Neutrophil Gelatinase-Associated Llipocalin

**Figure 1:**
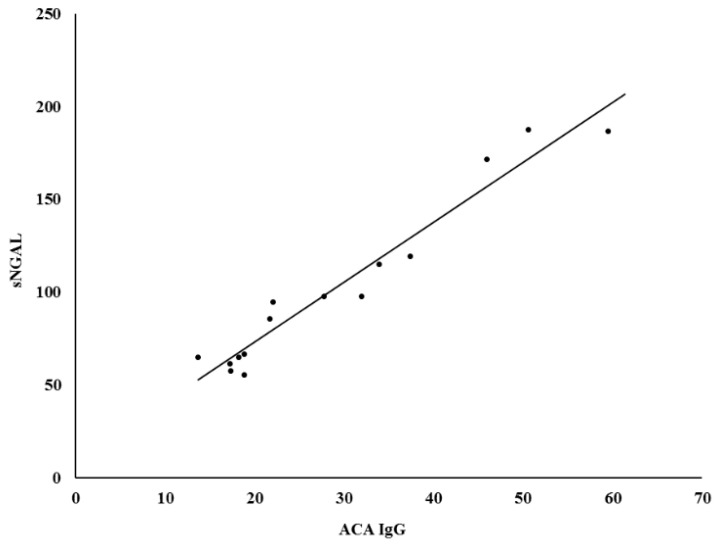
Correlation between sNGAL and ACA antibodies. sNGAL—serum Neutrophil gelatinase-associated lipocalin, ACA IgG—anticardiolipin antibodies type G.

**Figure 2:**
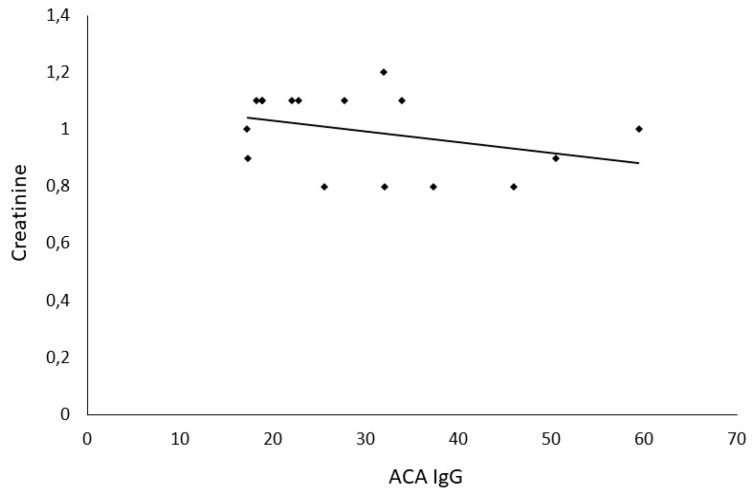
Correlation between creatinine and ACA antibodies. ACA IgG—anticardiolipin antibodies type G.

**Figure 3:**
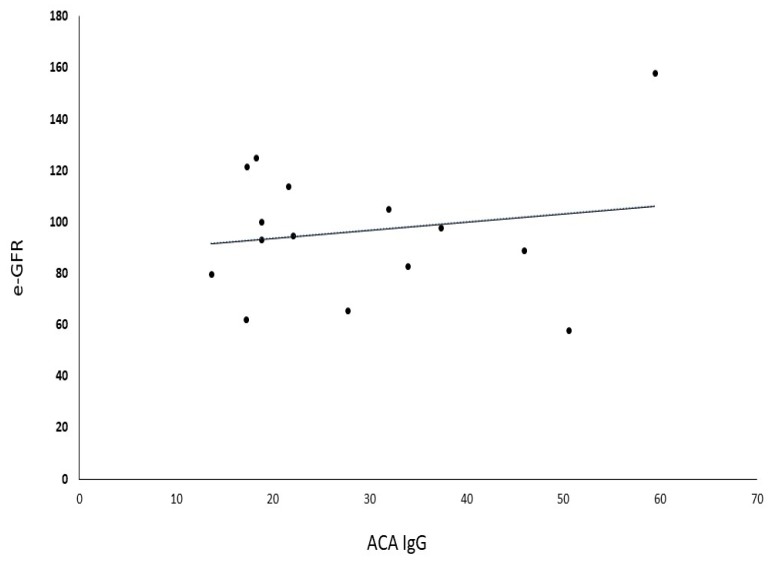
Correlation between e-GFR and ACA antibodies. e-GFR—estimated Glomerular Filtration Rate, ACA IgG—anticardiolipin antibodies type G.

The difference in sNGAL levels in patients with hypertension in comparison with the age-sex-matched control group (59.63 ± 41.5 ng/mL vs. 45.5 ± 21.5 ng/mL, *p* = 0.14) was not statistically significant. Moreover, sNGAL was found to be directly and positively correlated in patients with positive ACA IgG (r^2^ = 0.945, *p* < 0.0001) (Figure 1). No correlation was observed between aPL and sNGAL in control subjects (Table 4). Furthermore, no correlation was found between ACA and sCr in patients with positive ACA (r^2^ = 0.0175, *p* = 0.21) (Figure 2), as well as between ACA and e-GFR (r^2^ = 0.028, *p* = 0.34) (Figure 3).

## 4. Discussion

We report the clinical and laboratory findings of a cohort of 47 patients with hypertension who had positive tests for aPL. The diagnosis of APS is based on both clinical and laboratory criteria. Unfortunately, the criteria and the laboratory tests show wide variability within assays, making diagnosis quite difficult. It is well established that hypertension is a frequent clinical feature of APS which indicates a renal implication. The other clinical features include: renal artery stenosis (RAS), thrombotic microangiopathy (TMA), and other histological manifestations of the APS nephropathy (APSN). In the series of patients with hypertension that we studied, no patients developed any thrombotic manifestation of APS. Cacoub et al. described five patients with APS complicated by hypertensive crises, where all had normal magnetic resonance angiograms of the renal vessels. Three of their patients, according to contrast angiography, developed distal microangiopathy, but no stenosis or thrombosis of the main renal arteries [15]. Furthermore, non-thrombotic glomerulopathies are increasingly being described in patients with APS [16,17]. Fakhouri et al. studied kidney biopsies in patients with primary APS and glomerulonephritis and found predominantly non-thrombotic glomerulopathy in one-third of cases [18]. One of the most remarkable findings in this study is that statistically, a significantly elevated percentage of anti-b2GPI positive for type IgG were found in the hypertension group compared to controls. Our findings support those of other researchers who described non-thrombotic renal manifestation of APS.

Rollino et al. report in their study the frequency of aPL positivity at 8% [19]. Other studies report a prevalence of 1–5% for both ACL and lupus anticoagulant (LA) among control subjects [20]. The prevalence of aPL in children is more frequent. According to Avcin T et al., the estimated frequency of antibodies in children without any underlying disorder ranges from 3% to 28% [21].

The international consensus classification (ICS) for diagnosis of APS (Table 1) necessitates the presence of autoantibodies such as LA and/or IgG or IgM ACA in medium or high titer, and/or anti b2GPI (IgG and/or IgM) >99th percentile. This Abs detection should be persistent, defined as being present on two or more consecutive occasions with at least 12 weeks interval. However, it is not unusual to find patients with classical clinical manifestations of APS with negative aPL tests including ACA, ant b2GPI, and LA in daily clinical practice. To describe this syndrome, the term “seronegative APS” (SN-APS) has been suggested [20]. On the other hand, not all patients with aPL antibodies develop APS. These specific antibodies have been found in about 5% of the healthy population [22]. It has been hypothesized that nonpathogenic aPL are generated through immunological mechanisms such as nutritional antigens, mucosal immunity, and oxidative stress [23,24].

To estimate the renal function in our study we used different biomarkers such us sCr, e-GFR, and sNGAL. Importantly, the present study depicted that NGAL was found to be directly and positively correlated in patients with positive aPL. To our knowledge, there are no reports that analyze potential synergistic role of sNGAL and ACA as a diagnostic marker regarding the presence of early renal impairment in arterial hypertension.

Taken together, our results indicate that antiphospholipid antibodies may play an important role in hypertension and indicate renal involvement in APS. The early identification and appropriate therapeutic interventions in APS patients with kidney involvement may have important prognostic implications in preventing the determination of renal failure. We also suggest that it may be advisable for hypertensive patients suspected of APS to be tested for aPL antibodies, aiming for early and accurate diagnosis.

Finally, the lack of association between sNGAL levels with any measure of blood pressure limits the potential role of sNGAL as a predictive marker.

Potential limitations of our study were that we had a small sample size of patients. Moreover, we excluded patients with thrombotic events, which may be a clinical manifestation of APS. Moreover, in this study we do not measure other autoantibodies such as anti-Angiotensin II, anti-atrial natriuretic peptide or gene polymorphisms which may play role in the pathogenesis of hypertension [25,26].

## 5. Conclusions

Having considered all the above, and in agreement with recent literature [24,25], it seems that hypertension may be attributed to autoimmunity. It is well known that hypertension is prevalent in autoimmune disease and is an early symptom of the following renal involvement. Differences regarding positivity of aPL in the hypertension population and the sNGAL levels need further investigation and more data to overcome intra-laboratory variability and lack of standardization. Therefore, larger validation studies are still needed to validate and elaborate on our observations.

## Figures and Tables

**Table 1:** Diagnostic criteria of antiphospholipid syndrome [4]. The APS is present if at least 1 of the clinical criteria and 1 of the laboratory criteria that follow are met.

**Clinical Criteria**
(1) Vascular thrombosis 1 One or more clinical episodes of arterial, venous, or small-vessel thrombosis, in any tissue or organ. Thrombosis must be confirmed by objective validated criteria (i.e., unequivocal findings of appropriate imaging studies or histopathology). For histopathological confirmation, thrombosis should be present without significant evidence of inflammation in the vessel wall
(2) Pregnancy morbidity 2 (a) One or more unexplained deaths of a morphologically normal fetus at or beyond the 10th week of gestation, with normal fetal morphology documented by ultrasound or by direct examination of the fetus, or defined according to standard definitions (b) One or more premature births of a morphologically normal neonate before the 34th week of gestation because of: (i) eclampsia or severe preeclampsia or (ii) recognized features of placental insufficiency (c) Three or more unexplained consecutive spontaneous abortions before the 10th week of gestation, with maternal anatomical or hormonal abnormalities and paternal and maternal chromosomal causes excluded
**Laboratory Criteria**
(1) Lupus anticoagulant present in plasma, on 2 or more occasions at least 12 weeks apart, detected according to the guidelines of the International Society on Thrombosis and Haemostasis (Scientific Subcommittee on lupus anticoagulants/phospholipid-dependent antibodies) (2) aCL antibody of IgG and/or IgM isotype in serum or plasma, present in medium or at high titer (i.e., >40 GPL or MPL, or above the 99th percentile), on 2 or more occasions, at least 12 weeks apart, measured by a standardized ELISA (3) anti-b2GPI of IgG and/or IgM isotype in serum or plasma (at titers above the 99th percentile), present on 2 or more occasions, at least 12 weeks apart, measured by a standardized ELISA, according to recommended procedures.

**Table 2:** Clinical and demographic characteristics of patients with hypertension (cases) and controls.

	Patients	Controls	*p*
	*n* = 47	*n* = 21	
Age (years), mean (±SD),	65.9 ± 10.1	61 ± 11.3	0.1
Systolic BP mean (±SD), mm Hg	150.3 ± 17.1	124.7 ± 9.9	<0.0001
Diastolic BP mean (±SD), mm Hg	88.5 ± 11.5	80.7 ± 7.5	0.0068
Men (%)	22 (46.8)	8 (38.01)	
Body mass index mean (±SD), kg/m^2^	29.9 ± 6.4	29.5 ± 4.1	0.86
Diabetes mellitus (%)	14 (29.8)	1 (4.8)	<0.0001
History of coronary heart disease (%)	9 (14.1)	0	
Glucose mean (±SD) mg/dL	106.3 ± 69.3	96.7 ± 13.6	0.55
Urea mean (±SD) mg/dL	30.9 ± 11.3	30.5 ± 8	0.88
Creatinine mean (±SD) mg/dL	0.93 ± 0.28	0.86 ± 0.17	0.29
e-GFR, mean (±SD) ml/min/1.73m^2^	71 ± 21.6	87.6 ± 14.6	0.005
LDH mean (±SD) mg/dL	182.1 ± 63.9	177.1 ± 55.2	0.76
Total cholesterol means (±SD), mg/dL	189.8 ± 65.3	210.4 ± 0.18	0.18
Triglycerides means (±SD), mg/dL	152.9 ± 56.8	145.7 ± 52.6	0.79
HDL cholesterol means (±SD), mg/dL	41 ± 16.8	46.6 ± 8.6	0.16
Treatment			
Statins (%)	33 (70.2)	7(33.3)	
ACE Inhibitors (%)	22 (46.8)	-	
AT II (%)	22 (46.8)	-	
Diuretics (%)	4 (19.05)	-	
β—blockers (%)	10 (21.3)	-	
Calcium channel blockers (%)	10 (21.3)	-	

BP: blood pressure, e-GFR: estimated glomerular filtration rate, LDH: Lactate dehydrogenase, HDL: High density lipoprotein, ACE: Angiotensin converting enzyme, AT II: Angiotensin 2.

**Table 3:** Distribution of aPL between group I with hypertension (cases) and group II controls.

	Patients	Controls	*p*
	*n* = 47	*n* = 21	
aCL			
IgG mean (±SD)	12.16 ± 13.2	8.6 ± 9.9	0.32
IgM mean (±SD)	5.107 ± 4.01	2.6 ± 0.6	0.08
B2GPI			
IgG mean (±SD)	5.3 ± 5.9	4.42 ± 1.1	0.013
IgM mean (±SD)	5.1 ± 9.6	3.8 ± 1.6	0.17

**Table 4:** Correlation between aPL of the IgG and IgM Isotypes and sNGAL in control subjects.

Antibodies	Type	Kidney Injury Marker	r^2^	*p*
ACA	IgG	sNGAL	0.18	0.32
	IgM	sNGAL	0.21	0.23
β2GPI	IgG	sNGAL	0.11	0.61
	IgM	sNGAL	0.2	0.067
